# X-ray laser diffraction for structure determination of the rhodopsin-arrestin complex

**DOI:** 10.1038/sdata.2016.21

**Published:** 2016-04-12

**Authors:** X. Edward Zhou, Xiang Gao, Anton Barty, Yanyong Kang, Yuanzheng He, Wei Liu, Andrii Ishchenko, Thomas A. White, Oleksandr Yefanov, Gye Won Han, Qingping Xu, Parker W. de Waal, Kelly M. Suino-Powell, Sébastien Boutet, Garth J. Williams, Meitian Wang, Dianfan Li, Martin Caffrey, Henry N. Chapman, John C.H. Spence, Petra Fromme, Uwe Weierstall, Raymond C. Stevens, Vadim Cherezov, Karsten Melcher, H. Eric Xu

**Affiliations:** 1 Laboratory of Structural Sciences, Center for Structural Biology and Drug Discovery, Van Andel Research Institute, Grand Rapids, Michigan 49503, USA; 2 Center for Free Electron Laser Science, Deutsches Elektronen-Synchrotron DESY, 22607 Hamburg, Germany; 3 School of Molecular Sciences, and Center for Applied Structural Discovery, Biodesign Institute, Arizona State University, Tempe, Arizona 85287-1604, USA; 4 Department of Chemistry, Bridge Institute, University of Southern California, Los Angeles, California 90089, USA; 5 Department of Biological Sciences, Bridge Institute, University of Southern California, Los Angeles, California 90089, USA; 6 Joint Center for Structural Genomics, Stanford Synchrotron Radiation Lightsource, SLAC National Accelerator Laboratory, Menlo Park, California 94025, USA; 7 Linac Coherent Light Source (LCLS), SLAC National Accelerator Laboratory, Menlo Park, California 94025, USA; 8 Swiss Light Source at Paul Scherrer Institute, CH-5232 Villigen, Switzerland; 9 School of Medicine and School of Biochemistry and Immunology, Trinity College, Dublin D02 R590, Ireland; 10 Centre for Ultrafast Imaging, 22761 Hamburg, Germany; 11 Department of Physics, Arizona State University, Tempe, Arizona 85287, USA; 12 iHuman Institute, Shanghai Tech University, 2F Building 6, 99 Haike Road, Pudong New District, Shanghai 201210, China; 13 VARI-SIMM Center, Center for Structure and Function of Drug Targets, CAS-Key Laboratory of Receptor Research, Shanghai Institute of Materia Medica, Chinese Academy of Sciences, Shanghai 201203, China

**Keywords:** Biological physics, G protein-coupled receptors, Nanocrystallography

## Abstract

Serial femtosecond X-ray crystallography (SFX) using an X-ray free electron laser (XFEL) is a recent advancement in structural biology for solving crystal structures of challenging membrane proteins, including G-protein coupled receptors (GPCRs), which often only produce microcrystals. An XFEL delivers highly intense X-ray pulses of femtosecond duration short enough to enable the collection of single diffraction images before significant radiation damage to crystals sets in. Here we report the deposition of the XFEL data and provide further details on crystallization, XFEL data collection and analysis, structure determination, and the validation of the structural model. The rhodopsin-arrestin crystal structure solved with SFX represents the first near-atomic resolution structure of a GPCR-arrestin complex, provides structural insights into understanding of arrestin-mediated GPCR signaling, and demonstrates the great potential of this SFX-XFEL technology for accelerating crystal structure determination of challenging proteins and protein complexes.

## Background and Summary

Serial femtosecond X-ray crystallography (SFX) is an innovative development for protein structure determination, which uses X-ray free electron lasers (XFELs) as a radiation source to elicit diffraction from crystals^1^. An XFEL beam delivers extremely intense X-ray laser pulses that allow high resolution diffraction data collection from crystals of micrometer to nanometer size in random orientations. XFEL pulses of shorter than fifty femtosecond duration diffract protein crystals and terminate before significant radiation damage occurs in the protein, thus enabling data collection with reduced radiation damage using a dose higher than tolerable for cryogenically cooled crystals^2,3^. SFX has a high potential for structure determination of challenging proteins such as GPCRs and other membrane proteins that often don’t form crystals of sufficient size for synchrotron data collection^4^. Thus, SFX represents an important advancement in protein crystallography.

GPCRs comprise a large family of membrane proteins that are involved in many key signal transduction pathways in human physiology, and are targeted by approximately 40% of all approved pharmaceutical drugs^5–7^. Crystal structure determination of GPCRs and their complexes has been challenging due to their low expression levels, low stability in detergent micelles, and their recalcitrance to crystallization. In recent years, reports have been increasingly emerging about the successful structural determination by X-ray crystallography, owing to the development and the application of new techniques including protein fusion and the lipidic cubic phase (LCP) crystallization by which protein crystallogenesis occurs in a membrane-mimicking mesophase environment^8^. Crystallographic studies of GPCRs, nevertheless, remain a significant challenge because even in LCP many GPCRs produce micrometer-sized crystals that are too small for study at synchrotron sources.

In this paper, we report the deposition of the XFEL data for the recently published SFX structure determination of a rhodopsin-arrestin complex^9^ (Data Citation 1) as well as further details of crystallization, data collection, structure solution and validation. This rhodopsin-arrestin crystal structure is the first GPCR-arrestin complex structure that reveals the mechanism of GPCR recruitment of arrestin for desensitizing G protein signaling and initiating the arrestin-mediated signaling cascade.

## Methods

### Crystallization of rhodopsin-arrestin complex

Human rhodopsin and mouse visual arrestin-1 were used in our study. A T4 lysozyme (T4L)-rhodopsin-arrestin fusion protein was designed to form a stabilized rhodopsin-arrestin complex for crystallization with LCP technology. The fusion protein contains an N-terminal T4L (residues 1–162) and the full-length human rhodopsin (residues 1–348), which is followed by a 15-amino acid linker (AAAGSAGSAGSAGSA) and a mutant mouse visual arrestin (L374A, V375A, F376A, residues 10–392). This fused protein was expressed in HEK293S cells using a tetracycline-inducible expression vector with an N-terminal His8-MBP-MBP expression tag and a 3C protease cleavage site. The fusion protein was extracted from the cell membrane using an extraction buffer containing 0.5%(w/v) n-dodecyl-β-D-maltopyranoside (DDM, Anatrace) and 0.1% (w/v) cholesteryl hemisuccinate (CHS, Anatrace), and purified by amylose affinity chromatography. The protein sample was further purified using size exclusion chromatography and was concentrated to about 30 mg/ml for crystallization. *All-trans-*retinal at a molar ratio of 5 retinal:1 protein was added prior to crystallization.

**Figure 1:**
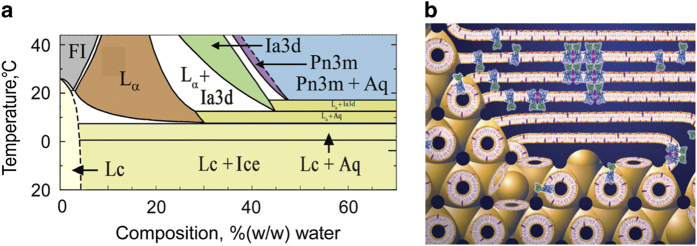
The lipidic cubic phase of 9.7 MAG facilitates the crystallization of T4L-rhodopsin-arrestin complex. (**a**) Temperature-composition phase diagram of 9.7 MAG/water system. The phase diagram was constructed based on small- and wide-angle X-ray scattering measurements made in the heating direction. Sample preparation and X-ray scattering measurements and analysis were as previously described^10,11^. The phases observed include the lamellar crystalline (Lc, or solid phase; yellow), the fluid isotropic (FI or liquid phase; grey), and the following liquid crystalline phases: lamellar liquid crystal (L_α_; brown), cubic-Ia3d (green) and cubic-Pn3m (purple). A separate aqueous phase observed in equilibrium with the solid or liquid crystalline phases is indicated by Aq. The phase diagram shows that the solid Lc phase stabilizes under equilibrium conditions below ~8 °C. The latter is some 10 °C below that observed with 9.9 MAG (monoolein)^11^ and is similar to what was found with 7.9 MAG^36^. This low solidification temperature enabled use in the current project of 9.7 MAG as a host lipid for LCP-SFX data collection in an evacuated sample chamber at 20 °C, where evaporative cooling created problems for measurements with 9.9 MAG but not with 7.9 MAG^14^. The maximum water carrying capacity of 9.7 MAG resides at ~50%(w/w) water which is considerably greater and smaller than that of 9.9 MAG^11^, and 7.7 MAG^37^, respectively. These observations indicate that the cubic mesophase of 9.7 MAG has larger aqueous channels compared to 9.9 MAG that are more like those of 7.7 MAG. This is consistent with 9.7 MAG supporting the growth of rhodopsin-arrestin-T4L crystals where the complex has sizable extra-membrane bulk best accommodated in a large aqueous channel. This parallels the observations made with 7.7 MAG as a host lipid for the β_2_AR-Gs complex structure determination^30^. (**b**) Cartoon representation of the T4L-rhodopsin-arrestin complex protein (rhodopsin is in blue, arrestin in green, and T4L is omitted) reconstituted in the LCP of 9.7 MAG, with the rhodopsin embedded in the bilayer of the LCP and the arrestin accommodated in the aqueous channel. Components of the precipitant solution are proposed to locally stabilize a lamellar domain into which the protein preferentially partitions from the LCP which acts as a reservoir. It is in the lamellar domain that nucleation and crystal growth take place (figure adapted from Li *et al.*^38^).

LCP crystallization of T4L-rhodopsin-arrestin fusion protein was performed using the monoacylglycerol (MAG) monopalmitolein (9.7 MAG, Nu Chek) containing 10% (w/w) cholesterol as the host lipid. Monoolein (9.9 MAG, Nu Chek), the most widely used host lipid for GPCR crystal growth, was first used as the host lipid for crystallization of the fusion protein with different concentrations of PEG 400 in combination with the StockOption Salt kit (Hampton Research) at various pH levels, but did not support crystal growth of the T4L-rhodopsin-arrestin complex. We thus tested several alternative lipids including 6.9 MAG (Nu Chek), 7.9 MAG (Anatrace), 9.7 MAG, 8.7 MAG (Anatrace) and 10.7 MAG (Nu Chek). Of all the lipids tested, only 9.7 MAG reproducibly facilitated the crystallization of the T4L-rhodopsin-arrestin complex. Figure 1a shows the temperature-composition phase diagram for 9.7 MAG constructed based on small- and wide-angle X-ray scattering measurements made in the heating direction^10,11^. The maximum water-carrying capacity of 9.7 MAG is close to 50% (w/w) at room temperature, which is considerably greater than that of 9.9 MAG (~40% (w/w)). This indicates that the cubic mesophase formed by 9.7 MAG has bigger aqueous channels compared to that of 9.9 MAG to better accommodate the relatively large non-membrane domains (arrestin and T4L) of the fusion protein and to facilitate the diffusion and crystallization of the fusion protein in the mesophase (Fig. 1b).

**Figure 2:**
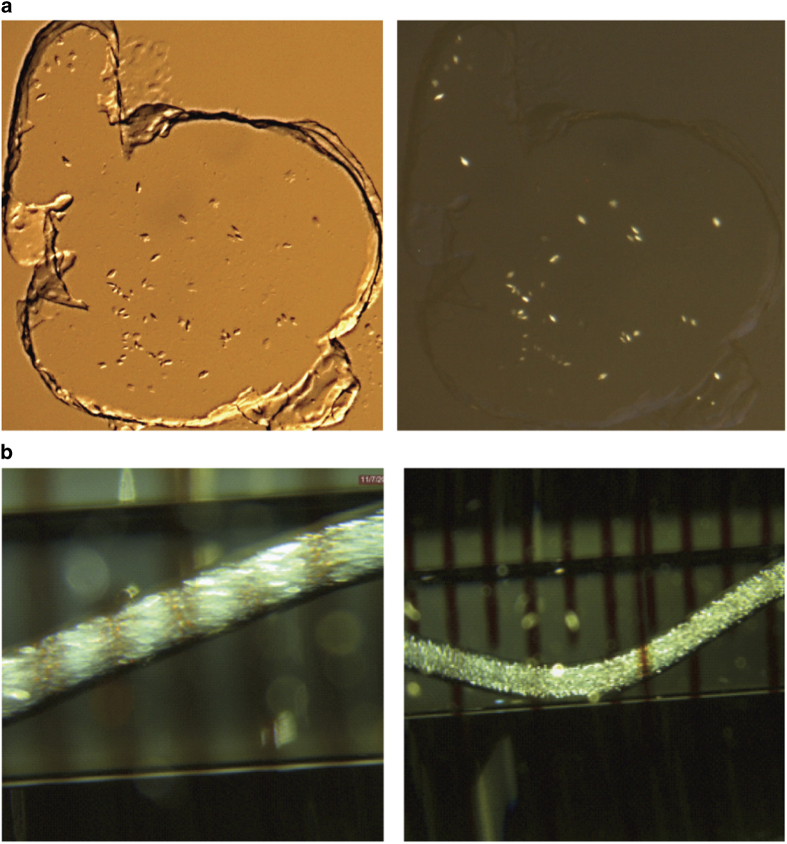
T4L-rhodopsin-arrestin crystals grown in LCP. (**a**) T4L-rhodopsin-arrestin crystals in sandwich plates viewed under bright field illumination (left panel) and polarized light (right panel). (**b**) Crystals in syringes under polarized light.

For initial LCP crystallization, a well-established protocol was used to reconstitute the T4L-rhodopsin-arrestin fusion protein into the lipid bilayer of the cubic phase^8^. Briefly, protein solution of about 30 mg/ml was mixed with 9.7 MAG containing 10% (w/w) cholesterol at a 1:1 ratio by weight using a coupled syringe mixer^10^ until a viscous transparent protein-laden homogenous LCP was formed. The crystallization was set up using a Gryphon LCP robot (Art Robbins Instruments) or an NT-8 LCP robot (Formulatrix). A volume of 50 nl boluses of LCP were applied to each well of a 96-well glass sandwich plate (Molecular Dimensions or Marienfeld-Superior), covered with 0.8 μl crystallization solutions and sealed with a glass cover slide. The sandwich plates were kept at 20 °C, and multiple initial hits were identified after a few days from home-made crystallization screens, which were prepared using 30% (v/v) PEG 400 in combination with 100 or 400 mM salts from the StockOptions Salt kit and buffers of pH 5, 6, 7, and 8 (refs. 9, 12). The final optimized crystals with sizes of 5–20 μm were obtained from a precipitant containing 28% PEG 400, and 50 mM magnesium acetate, 50 mM sodium acetate at pH 5.0. These crystals were harvested directly from LCP using MiTeGen loops, frozen in liquid nitrogen and used for synchrotron data collection (Fig. 2a).

SFX data collection requires tens to hundreds μl of LCP filled with microcrystals at high density. We therefore used our previously developed protocols to scale-up crystallization set-up using 100 μl gas-tight Hamilton syringes^4,13^. Briefly, a volume of 5 μl protein-laden LCP, as used for crystallization in sandwich plates, was slowly injected as a continuous string into a 100 μl syringe filled with 60 μl of crystallization solution^10^. High density microcrystals grew in the mesophase in the syringes over 12 to 24 h at 20 °C. The best crystals were obtained from the crystallization condition of 32%(v/v) PEG 400, and 150 mM ammonium phosphate at pH 6.4. The crystal sizes were at about 5–10 μm measured using a polarized light microscope (Fig. 2b). The LCP with crystals was consolidated from several syringes and transferred into the LCP injector^14^ for XFEL diffraction data collection.

### Synchrotron diffraction data collection and processing

**Figure 3:**
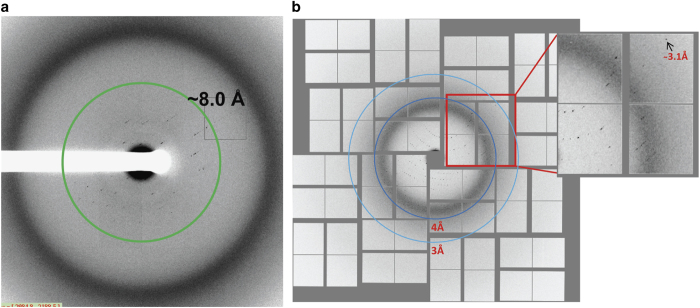
X-ray diffraction patterns of T4L-rhodopsin-arrestin crystals. (**a**) A diffraction image of a T4L-rhodopsin-arrestin crystal collected at LS-CAT of APS. The green ring indicates the position of reflections at 8.0 Å resolution; (**b**) an XFEL diffraction image collected at LCLS with two blue rings indicating 3.0 and 4.0 Å resolution reflections, respectively.

Data sets to about 8.0 Å resolution were collected from multiple crystals (5–10 crystals) using the 21-ID-D beam line of LS-CAT with a Mar 300 CCD detector or 23-ID-D beam line of GM/CA-CAT with a Pilatus 6 M detector at the Advanced Photon Source (APS), Argonne National Laboratory at Argonne, IL (Fig. 3a). An additional data set to 7.7 Å was collected from a single crystal of about 20 μm size using a 10 μm beam of 1.033 Å wavelength and 0.1 s exposure time per 0.1° oscillation with a Pilatus 6 M pixel detector at a distance of 600 mm at the X10SA beam line of the Swiss Light Source. The diffraction data were reduced, integrated and scaled with XDS^15^. The data statistics are shown in Table 1. While the 7.7 Å data set from a single crystal was used for twinning analysis and the validation of the structure model from SFX data, all other synchrotron data sets were not used for structure determination of the T4L-rhodopsin-arrestin complex because of their lower resolution.

### X-ray free electron laser data collection

The SFX experiments were performed using the LCLS Coherent X-ray Imaging (CXI) instrument at the SLAC National Accelerator Laboratory (Menlo Park, California, USA). Rhodopsin-arrestin complex crystals of 5–15 μm in LCP were streamed across the XFEL beam in a continuous mesophase column at a flow rate of 0.18 μl/min using an LCP injector^14^ with a 50 μm diameter nozzle. X-ray pulses of 48 fs duration and 9.5 keV photon energy (1.3 Å wavelength) were focused to a spot of ~1 μm FWHM using Kirkpatrick-Baez mirrors and centered on the LCP column using an inline microscope. A transmission of 3–10% was used and an average flux of 3×10^10^ photons per pulse was delivered to single crystals in the LCP column with an estimated maximum dose of about 25 MGy per crystal. Crystals in the LCP stream were randomly oriented in the interaction region, producing a crystal diffraction pattern whenever the regular 120 Hz X-ray pulse repetition rate happened to coincide with a crystal being in the focal region. Diffraction patterns were read out and recorded with a Cornell-SLAC pixel array detector (CSPAD)^16^ at a sample-to-detector distance of 100 mm after each X-ray pulse (Fig. 3b and Data Citation 2). Approximately 100 μl of crystal-laden mesophase was used for data collection.

Three slightly different preparations of rhodopsin-arrestin complex were used: batches 1, 2, and 3, these were noted internally as Rho-Arr-ATR, Rho-Arr(C234)-ATR, and Rho-Arr(C235)-ATR-IP6, corresponding to runs 2–50, 52–64 and 69–73, respectively of experiment LE79 at the CXI instrument at LCLS in November 2014 (Data Citation 2). Even though all three batches were expected to be isomorphous, we found during data processing that merging data from batches 1+2 (runs 2–64) produced superior statistics and electron density than merging all three batches (runs 2–73) (Data Citation 2), despite the smaller number of crystals included in the data set consisting of only batches 1+2 compared to all data. We speculate that this is due to slight non-isomorphism between the different individual preparation conditions. All data has been deposited with the CXIDB (see the section of ‘Data records’, and Data Citation 2) even though only data from batches 1+2 were used for the published structure.

### XFEL data processing

About 5 million data frames were recorded in a 10-hour data acquisition period using crystal sample batch 1+2. The initial data were reduced and analyzed using the program Cheetah^17^. Of the recorded frames, 22,262 images were identified containing potential crystal hits with more than 40 Bragg peaks of greater than one pixel in size and a signal-to-noise ratio better than 6 after local background subtraction. This represented a hit rate of about 0.45% (Fig. 3b).

Data has been deposited with the CXIDB as both processed hits and raw data. Hits found by Cheetah are saved in Cheetah's single-frame HDF5 format with the minimum number of corrections necessary to make the data useable: specifically, detector dark correction and common mode correction using unbonded pixels on the cspad detector, with saturated and bad pixels flagged in a separate mask saved along with the data (saveDetectorCorrected option). We have additionally deposited the raw data for the whole experiment in the CXIDB enabling the detector correction and hit finding steps to be repeated if desired. This raw data is deposited in HDF5 format created using LCLS HDF5 file translation in which raw data is saved using the layout documented in the LCLS online documentation, enabling processing without the need to install LCLS-specific XTC file readers. A run table is included to aid analysis of the raw data.

**Figure 4:**
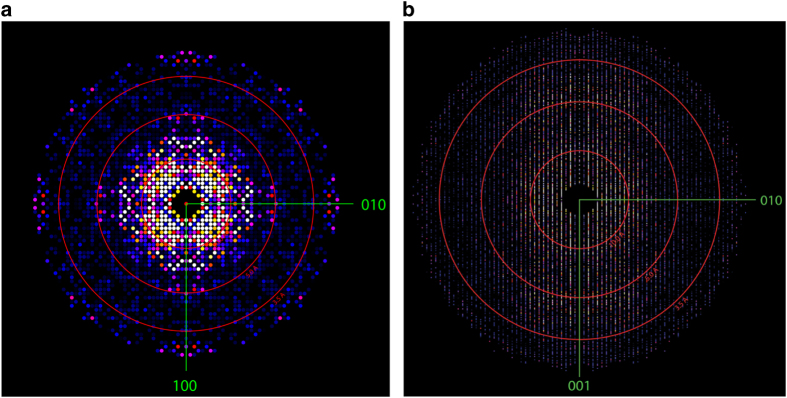
X-ray free-electron laser composite diffraction patterns of T4L-rhodopsin-arrestin crystals. The reflection intensity of the T4L-rhodopsin-arrestin crystals is displayed as 2D maps at the central section perpendicular to the unit cell *c* (panel **a**) and *a* axes (panel **b**), respectively. Resolution rings at 10.0, 5.0, and 3.5 Å are shown.

Data frames with crystal hits were extracted in HDF5 format for further analysis with the program package CrystFEL^18^. These potential crystal hits prior to indexing have been deposited with the CXIDB (CXIDB ID 32, Data Citation 2) and are the subject of this Data Descriptor. Of the potential crystal hits, 18,874 diffraction patterns were identified and indexed using the program ‘indexamajig’ in the CrystFEL package with a combination of indexing methods of MOSFLM^19^, XDS^15^ and DirAx^20^. Reflections were integrated over the three-dimensional reflection profile using ‘process-hkl’ by which the final integrated Bragg intensities were constructed with partially recorded intensities from single shot diffraction patterns of randomly oriented single crystals. An integration region radius of two pixels was used to avoid overlaps with neighboring peaks due to the high spot density resulted from the large unit cell dimensions. All merged intensities can be visualized by plotting as a precession-style image along the [001] and [100] axes of the reciprocal space (Fig. 4a,b). The crystals appeared to be tetragonal or very close to tetragonal, with an apparent reflection condition of l=4n (n is an integer) for the 00 l reflections and a unit cell of a=b=109.2 Å, c=452.6 Å, and α=β=γ=90°. The diffraction strength was anisotropic and the data was truncated using the CrystFEL program ‘get_hkl’ to 3.3 Å along the c* axis and 3.8 Å along the a* and b* axes based on the correlation coefficient statistics (CC*) of the data^21^ (Table 1). This anisotropy in resolution can be seen in the zone axis sections (Fig. 4a,b).

**Figure 5:**
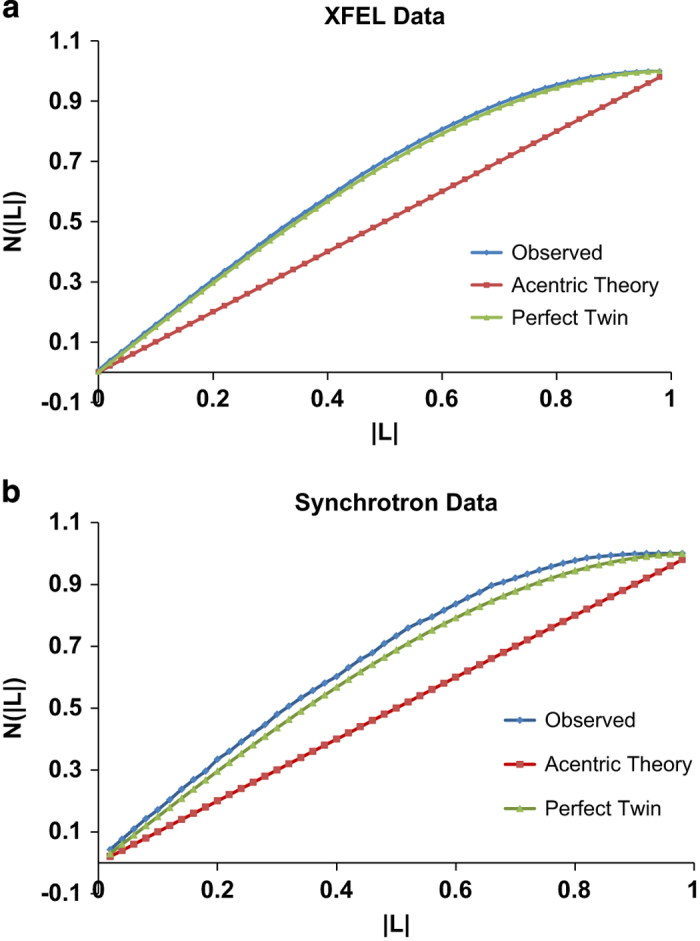
L-test plots of diffraction data. (**a**) L-test of the XFEL data from T4L-rhodopsin-arrestin crystals, and (**b**) L-test of the synchrotron data from a single crystal, both indicating nearly perfect twinning of the crystals.

For certain merohedral space groups, an indexing ambiguity arises which results in a ‘computationally twinned’ dataset when data from many crystals are merged together in serial crystallography^17^. An L-test^22^ performed using Phenix.xtriage^23^ showed a mean |L| of 0.399, a mean L^2^ of 0.191 that suggested the presence of perfect twinning in our XFEL data (Fig. 5a). Several attempts using recently reported methods^24,25^ to resolve the indexing ambiguity failed, indicating that the crystals were probably physically twinned. To confirm that the perfect twinning of the data was due to the physically twinned crystals, an L-test of the synchrotron data collected from a single crystal was carried out. The L-test revealed the mean |L| and the mean L^2^ of the synchrotron data were 0.342 and 0.169, respectively, and the same twin law as the XFEL data (Fig. 5b), confirming that the perfect twinning of our XFEL data was indeed due to the twinned crystals, which could be either in a tetragonal space group and merohedrally twinned, or in an orthorhombic space group and pseudo-merohedrally twinned because of their nearly identical unit cell axes of a and b and non-crystallographic rotational symmetry^26,27^. The exact space group was later determined by Zanuda^28^.

### Structure determination

**Figure 6:**
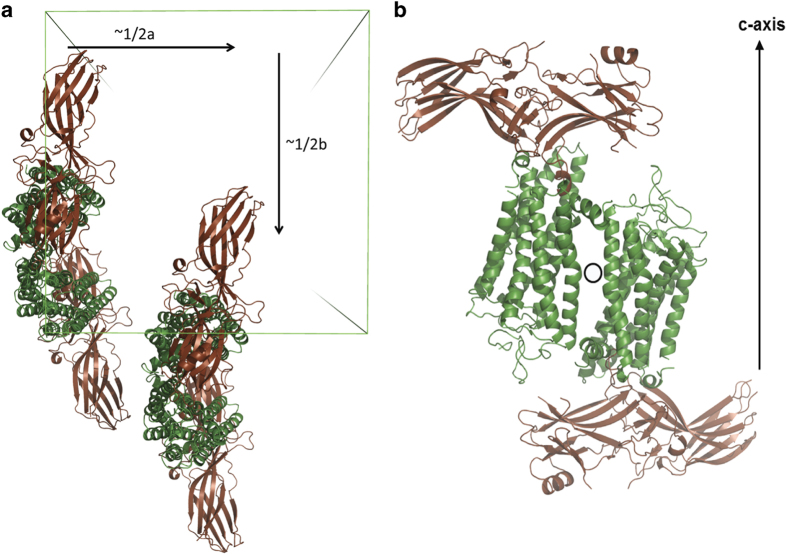
Pseudo symmetries of rhodopsin-arrestin crystal packing. (**a**) Pseudo-translational symmetry of the four T4L-rhodopsin-arrestin complex molecules is shown in the asymmetric unit with two of the four molecules translated by approximately half of the unit cell along the unit cell *a* and *b* axes. The unit cell is shown as a square box. (**b**) Pseudo-rotational symmetry of T4L-rhodopsin-arrestin complex molecules is shown in the asymmetric unit, where two of the four molecules can be overlaid by a 180° rotation around the *a/b* axis. Crystals with pseudo rotational symmetry are prone to twinning and the data is consistent with pseudomerohedral twinning of *P*2_1_2_1_2_1_.

For structure determination, molecular replacement (MR) was performed using available structural models of G protein peptide-bound rhodopsin (PDB code 4A4M^29^), the pre-activated arrestin (PDB 4J2Q^5^) and T4 lysozyme (PDB 3SN6 (ref. 30)), and the diffraction data that was expanded to Laue group 4/m. MR searches were carried out in all possible space groups of the tetragonal system using the program Phaser^31^. Four copies of either rhodopsin or arrestin and three copies of T4L were found in space group *P*4_3_ with Z scores greater than 8 for each solution. There is a pseudo-translational symmetry by ~1/2a and 1/2b, and a pseudo-rotational symmetry nearly parallel to the twin operation (k, h, −l) of the molecules in the asymmetric units (Fig. 6a,b).

Because of the perfect twinning and the presence of pseudo-symmetry elements, the apparent space group of a crystal may not be its true space group. We therefore analyzed the data with the structural model obtained from space group *P*4_3_ using Zanuda^28^ (Table 2), and found that the space group of *P*2_1_2_1_2_1_ gave the best Z score and free R values, and was more likely the true space group for this data set. The data was then expanded to the Laue group of mmm and MR solutions were found in space group *P*2_1_2_1_2_1_ with better statistics (Table 2). Those results indicated that the true space group of the crystals was *P*2_1_2_1_2_1_, and the crystals appeared to be in space group *P*4_3_ due to the pseudo-merohedral twinning, caused by the very close a and b axes of the lattice and the non-crystallographic rotational symmetry that corresponds to the twin operator k,h,−l. This physical twinning, which corresponds to the same transformation as the only possible indexing ambiguity for these unit cell parameters and space group assignment, explains why our attempts to resolve the indexing ambiguity did not succeed.

The structural model from MR was initially rebuilt and refined against the XFEL data without the twin law using COOT^32^ and Phenix^23^. Composite omit maps calculated using a Phenix program were used to guide manual building of the loop regions missing in the original models, and to rebuild misplaced residues. After many iterated cycles of model building and refinement, the R_free_ reduced, but could not be further improved beyond 36%, which suggested a point to apply twin law for further refinement. Further fine-tuning refinement was done using the methods of individual position, group B-factor and TLS with NCS restraints and twin law. The final R_work_ was 25.2% and the R_free_ was 29.3%, which demonstrated that the model was correctly built and refined (Table 3). The final model included four copies of rhodopsin-arrestin complex, two copies of full T4 lysozyme in complex A and D, respectively, and a partial T4 lysozyme molecule (residues 2–12 and 58–161) in complex C. The rhodopsin ligand *all-trans* retinal was not built in the model because of weak density.

### Structure validation

**Figure 7:**
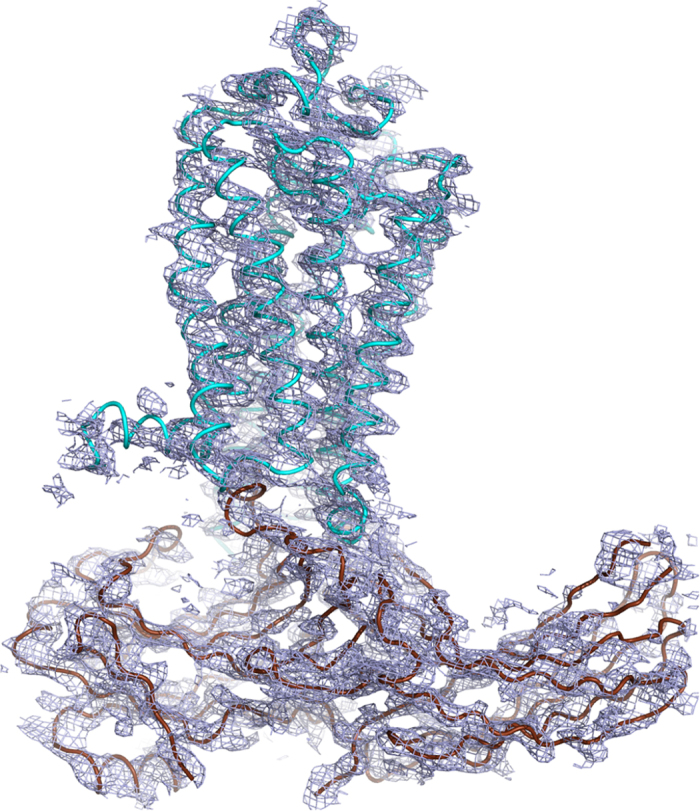
A simulated-annealing 2Fo-Fc composite omit map. The map was calculated from the 3.8 Å/3.8 Å/ 3.3 Å XFEL data with 3,000 K simulated annealing and was contoured at 1 σ. The simulated-annealing omit map supports the overall arrangement of the rhodopsin-arrestin complex. The complex structure is shown with rhodopsin colored in cyan, arrestin in brown and T4L is not shown in the map.

**Figure 8:**
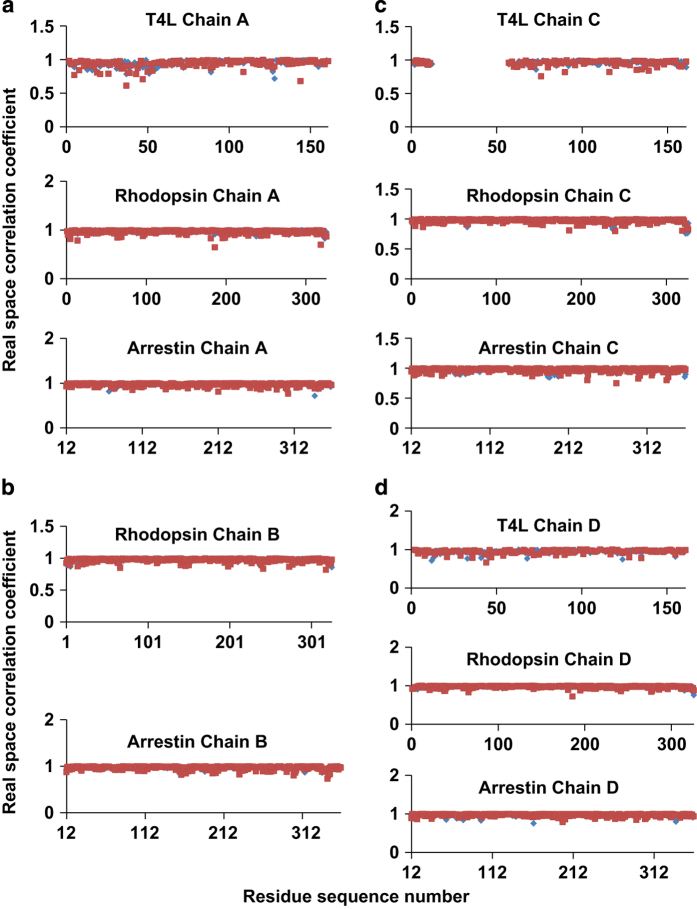
The real space correlation coefficients between the structure and a 2mFo-DFc map. The real space correlation coefficients of all the residues in the model were calculated against a 2mFo-DFc map using the CCP4 EDSTATS program. Plots a-d are for T4L-rhodopsin-arrestin complexes A, B, C, and D, respectively, with main chain residues in blue and side chain residues in red.

**Figure 9:**
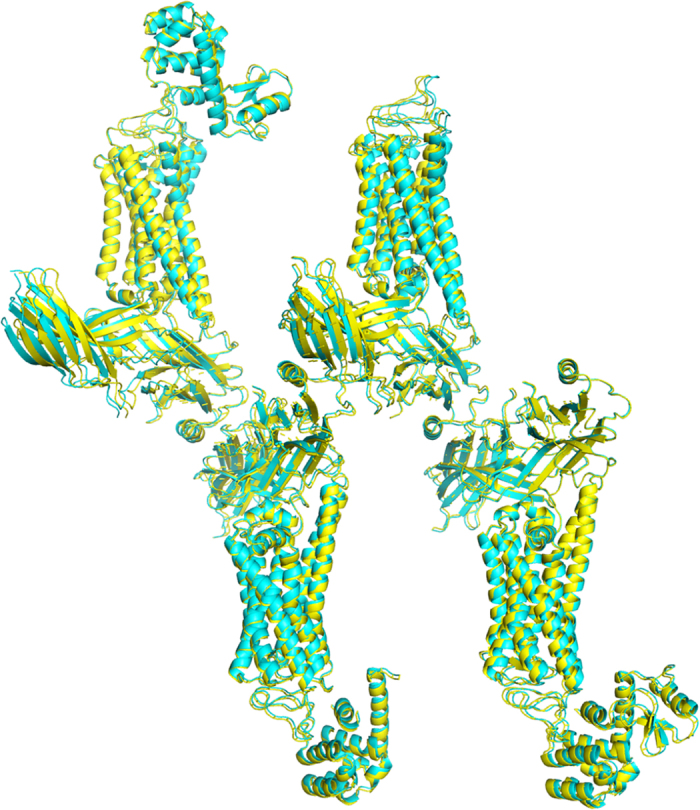
The superposition of the structure models from synchrotron (yellow) and XFEL data (cyan).

**Figure 10:**
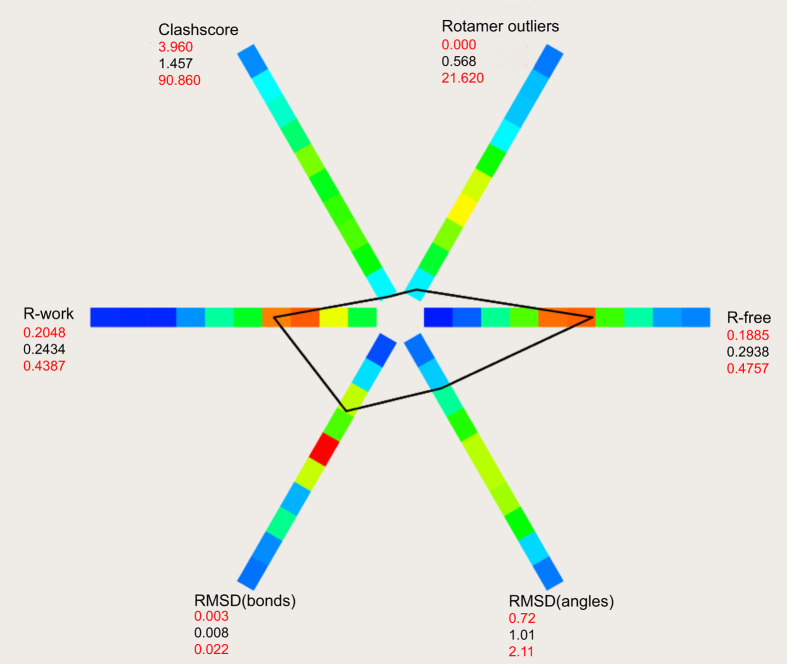
POLYGON analysis of the quality statistics of the rhodopsin-arrestin complex structure. A comparison of the rhodopsin-arrestin complex structure with 535 structures in the PDB at comparable resolution indicates that the quality of the current structure is equal to or better than most X-ray structures in the database of similar resolution.

The structural model from SFX was extensively validated using various independent biochemical and biophysical methods, including electron microscopy, double electron-electron resonance spectroscopy, hydrogen-deuterium exchange mass spectrometry, cell-based rhodopsin-arrestin interaction assays, and site-specific disulfide cross-linking experiments as reported in the original paper^9^. Here we focus on the crystallographic validation of the structure. A composite omit map calculated using a Phenix^23^ program with simulated-annealing at 3,000 K showed a density of good quality which suggested that the model was correctly built (Fig. 7). Real space correlation coefficients against a 2mFo-DFc map for each chain of the model calculated using the EDSTATS program in CCP4 (ref. 33) indicated an overall good fit between the structural model and the electron density map (Fig. 8a–d). To further validate the structural model from SFX, we placed the model in the asymmetric unit of the synchrotron data and performed rigid body and group B-factor refinement with twin law and NCS restraints. The refined model with good R factors (R_work_=28.5%, R_free_=33.5%, Table 3 and Data Citation 3) against the synchrotron data could be superposed on the XFEL model with only slight difference (Fig. 9), which confirmed that the XFEL data was as reliable as a synchrotron data set for crystal structure determination. The MolProbity analysis revealed an all-atom clash score of 1.47, 0.59% rotamer outliers, 100% favorable and allowed Ramachandran regions, and an overall MolProbity score of 1.13, corresponding to a better than average model quality compared to those of similar resolution from the PDB database. The structure was also analyzed using POLYGON in Phenix^23^, which indicated that the quality of the model statistics was above average compared with 535 entries of similar resolution in the PDB (Fig. 10).

## Data Records

All data records listed in this section are available at the cxi database (Data Citation 2), and are accessible via web service at http://www.cxidb.org with CXIDB ID 32.

## Technical Validation

The technology of serial femtosecond X-ray crystallography using X-ray free electron lasers has been developed and validated by several pioneer groups^1,4,34,35^. The crystal structure of the rhodopsin-arrestin complex determined using the XFEL dataset was extensively validated through multidisciplinary technologies including HDX, DEER and disulfide crosslinking as described in the original paper^9^. The XFEL data was also validated by crystallographic analysis as described in the section of Structure Validation. These validations support the technical quality of the XFEL dataset to be used for 3-dimentional structure determination.

In summary, obtaining the crystal structure of the rhodopsin complex faced many challenges ranging from protein engineering to formation of a stable protein complex, from crystallization to data collection and processing, and from structure determination to validation by multiple inter-disciplinary techniques. The structure determination of the rhodopsin-arrestin complex using XFEL data provides an important example that demonstrates the great potential of this technology for solving crystal structures of challenging proteins that do not grow crystals of sufficient size for crystallographic studies using conventional X-ray sources.

## Additional Information

**How to cite this article:** Zhou, X. E. *et al.* X-ray laser diffraction for structure determination of the rhodopsin-arrestin complex. *Sci. Data* 3:160021 doi: 10.1038/sdata.2016.21 (2016).

## Supplementary Material



## Figures and Tables

**Table 1:** Diffraction data collection and processing.

**Data collection**	**XFEL**	**Synchrotron**
PDB code	4ZWJ (Data Citation 1)	5DGY (Data Citation 3)
Wavelength (Å)	1.33	1.03
Space group	*P*2_1_2_1_2_1_	*P*2_1_2_1_2_1_
Cell dimensions		
*a*, *b*, *c* (Å)	109.2, 109.2, 452.6	107.3, 107.3, 460.3
σ, β, γ (°)	90, 90, 90	90, 90, 90
Resolution (Å)	31.5–3.3 (3.42–3.30)*	30–7.7 (8.1–7.7)
*R*_split_ or R_merge_ (%)	19.1 (58.7)	31.4 (118.8)
SNR or I/σI	4.7 (1.7)	3.79 (1.08)
CC*	0.996 (0.87)	1.00 (0.81)
Completeness (%)	76.3 (6.4)	97.5 (90.8)
Multiplicity	383 (116)	4.68 (4.12)
This table was adopted from the Supplementary Materials of the original paper^9^ with addition of the statistics of the synchrotron data collected at X10SA beamline of the Swiss Light Source.		

*Values in parentheses are for highest-resolution shell.

**Table 2:** Zanuda analysis.

**Space group**	**Cell (Å)**	**Nmol/asu**	**TFZ**	**R**_**work**_ **(%)**	**R**_**free**_ **(%)**
*P*4_3_	*a*=*b*=109.2, *c*=452.6	4	23.9	30.7	36.9
*P*2_1_2_1_2_1_	*a*=*b*=109.2, *c*=452.6	4	1.7	25.1	31.6
*P*2_1_	*a*=*b*=109.2, *c*=452.6	8	27.8	23.4	31.8
C2	*a*=*b*=109.2, *c*=452.6	4	10.0	44.6	47.8
C2	*a*=*b*=154.5, *c*=452.6	8	14.5	30.9	35.7
This table was adopted from the Supplementary Materials of the original paper^9^ with addition of the data and model statistics of the synchrotron data.					

**Table 3:** Refinement and model statistics.

**Refinement**	**XFEL**	**Synchrotron**
PDB code	4ZWJ (Data Citation 1)	5DGY (Data Citation 3)
Resolution range (Å)	31.0–3.3 (3.4–3.3)*	30–7.7 (8.1–7.7 )*
No. reflections (all/free set)	62,613/3,098	6630/845
*R*_work_/*R*_free_ (%)	25.2/29.3 (33.3/40.8)*	28.5/33.5 (30.2/33.7)*
No. atoms		
Rhodopsin	10,344	10,344
Arrestin	10,840	10,840
T4L	3,481	3,481
B factors		
Wilson	112.0	303
Proteins	159.7	420
R.M.S. deviations		
Bond lengths (Å)	0.008	0.008
Bond angles (°)	1.0	1.0
Ramachandran		
Favored (%)	96.3	96.3
Outliers (%)	0.0	0.0
Clash Score	1.47	1.53
MolProbity score	1.13	1.14
This table was adopted from the Supplementary Materials of the original paper^9^ with addition of the model statistics of the synchrotron data collected at X10SA beamline of the Swiss Light Source.		

*Values in parentheses are for highest-resolution shell.
